# Maternal Separation Stress Affects Voluntary Ethanol Intake in a Sex Dependent Manner

**DOI:** 10.3389/fphys.2021.775404

**Published:** 2021-12-07

**Authors:** Natalia Bonetti Bertagna, Cristiane Aparecida Favoretto, Ben Tagami Rodolpho, Paola Palombo, Thais Suemi Yokoyama, Thamires Righi, Cássio Morais Loss, Rodrigo Molini Leão, Tarciso Tadeu Miguel, Fábio Cardoso Cruz

**Affiliations:** ^1^Molecular and Behavioral Neuroscience Laboratory, Department of Pharmacology, Federal University of São Paulo, São Paulo, Brazil; ^2^Pharmacology Laboratory, Department of Pharmacology, Biomedical Sciences Institute, Federal University of Uberlândia, Uberlândia, Brazil; ^3^National Institute for Translational Medicine (INCT-TM), National Council for Scientific and Technological Development (CNPq/CAPES/FAPESP), Ribeirão Preto, Brazil

**Keywords:** maternal separation, stress, ethanol intake, sex differences, extended amygdala

## Abstract

Maternal separation (MS) stress is a predictive animal model for evaluating the effects of early stress exposure on alcohol use disorders (AUD). The extended amygdala (AMY) is a complex circuit involved in both stress- and ethanol-related responses. We hypothesized that MS stress may increase ethanol consumption in adulthood, as well as augment neuronal activity in extended AMY, in a sex-dependent manner. We aimed to investigate the influence of MS stress on the ethanol consumption of male and female mice, and the involvement of extended amygdala sub-nuclei in this process. The C57BL/6J pups were subjected to 180min of MS, from postnatal day (PND) 1 to 14. The control group was left undisturbed. On PND 45, mice (*n*=28) in cages were exposed to a bottle containing 20% ethanol (w/v) for 4h during the dark period of the light-dark cycle, for 3weeks. Afterward, mice underwent ethanol self-administration training in operant chambers under fixed ratio (FR) schedule. Then, subjects were tested under 2h sessions of a progressive-ratio (PR) schedule of reinforcement (the last ratio achieved was considered the breaking point), and at the end, a 4h session of FR schedule (binge-intake). An immunohistochemistry assay for Fos protein was performed in Nucleus Accumbens (NAcc), Bed Nucleus of Stria Terminalis (BNST), and AMY. Our results showed that in the third week of training, the female MS group consumed more ethanol than the respective control group. The MS group presented increased breakpoint parameters. Female control group and male MS group were more resistant to bitter quinine taste. Increased Fos-immunoreactive neurons (Fos-IR) were observed in the central nucleus of AMY, but not in NAcc nor BNST in male maternal-separated mice. Maternal separation stress may influence ethanol intake in adulthood, and it is dependent on the sex and reinforcement protocol.

## Introduction

Alcohol is the most commonly used substance of abuse worldwide, and about 2.3 billion people aged 15–16years old have used this substance at least once in their life (WHO, 2018). Harmful use of alcohol brings many social and economic impairments to individuals (UNODC, 2020). Moreover, this harmful use is considered one of the most important risk factors for public health, as it is estimated that 3 million (5.3%) global deaths are attributed to alcohol intake (WHO, 2018).

Alcohol use disorders (AUD) are among the most prevalent mental disorders (American Psychiatric Association, 2013; Rhem and Shield, 2019). It is characterized by an inappropriate pattern of alcohol use, in which individuals exhibit compulsive heavy alcohol consumption, and loss of control over alcohol intake, despite adverse consequences involving their health, family, social life, and occupational activities (American Psychiatric Association, 2013; Connor et al., 2016; Carvalho et al., 2019). Epidemiological studies have shown that AUD is more prevalent in men compared to women (WHO, 2018; Rhem and Shield, 2019). In this sense, studies have shown that 237 million men and 46 million women around the world are diagnosed with AUD (WHO, 2018).

Stress is considered an important environmental risk factor for the development of alcohol and other substance use disorders, such as cocaine, amphetamine, and opiates (Piazza and Le Moal, 1998; Sinha, 2008; Camarini et al., 2018; Mukhara et al., 2018). Stress may be related to initiation, maintenance, escalation, and relapse of drug use (Piazza and Le Moal, 1998; Sinha, 2008; Camarini et al., 2018; Mukhara et al., 2018). However, the relationship between stress exposure and alcohol intake is complex and poorly understood (Becker et al., 2011; Noori et al., 2014).

Early life exposure to stress, such as sexual abuse, violence, and negligence can promote immediate and long-lasting neuroendocrine and behavioral changes (Caldji et al., 2000; Meaney, 2001; Enoch, 2011; Nishi et al., 2013). These changes may include hypothalamic-pituitary-adrenal (HPA) axis alterations and morphological changes in the brain associated with the development of psychopathologies, including AUD (Meaney, 2001; Sinha, 2001, 2008; Miczek et al., 2008; Enoch, 2011; Novick et al., 2018). For instance, Tarullo and Gunnar (2006) reported that childhood mistreatment provoked alterations on HPA axis development, leading to low basal levels of cortisol in adulthood. It is also observed a smaller volume of hippocampus, amygdala (AMY) hyperactivation, and delayed pre-frontal maturation in adult that underwent early mistreatment (Teicher et al., 2003; Rao et al., 2010).

Newborn rodents depend on maternal care in order to achieve appropriate nutrition, thermoregulation, and protection against predators. In addition, it can provide long-lasting effects on the brain, and decreased reactivity to stress (Novick et al., 2018). In the MS protocol, pups are separated daily from their dam, usually from post-natal day (PND) 1 to 14, for short or long periods (Cruz et al., 2008; Novick et al., 2018). Prolonged periods of MS (180min) have been shown to be an intense stressor for the litter, and can negatively impact maternal care during behavioral, physiological, and neuroendocrine maturation (Levine, 1967).

Several studies have demonstrated that MS stress in rodents increased alcohol consumption during adulthood, in different paradigms, such as two-bottle choice and operant self-administration (Huot et al., 2001; Ploj et al., 2003; Roman and Nylander, 2005; Kawakami et al., 2007; Cruz et al., 2008; Portero-Tresserra et al., 2018). In addition, male mice submitted to long periods of MS stress were more susceptible to the effects of chronic alcohol administration on plasma corticosterone levels (Kawakami et al., 2007).

The MS stress seems to produce sex-dependent effects. Roman et al. (2005) showed that alcohol intake of stressed male ethanol-preferring Alko Alcohol (AA) rats was higher than the control group consumption. However, stressed female AA rats had a reduced alcohol intake and preference relative to controls. A study conducted by Kawakami et al. (2007) also showed that prolonged maternal separation (MS) induced a faster behavioral sensitization in females compared to male mice.

Extended AMY, which consists of the Nucleus Accumbens shell (NAcc shell), Central Amygdala (CeA), and Bed Nucleus of Stria Terminalis (BNST), is complex neural circuitry that is known especially for its role in negative affects during ethanol withdrawal (Koob and Volkow, 2010; Koob, 2013). Studies show that the extended amygdala is involved in the modulation of stress responses and AUD (for review, see Centanni et al., 2019). For instance, it has been demonstrated that an acute alcohol systemic injection increases CeA and BNST c-fos expression (Sharko et al., 2016; Saalfield and Spear, 2019). In the same sense, Leriche et al. (2008) also demonstrated that intragastric alcohol administration was able to augment c-fos expression in subregions of NAcc, AMY and BNST. Furthermore, Anstrom et al. (2009) showed that social defeat stress increased dopamine release in NAcc of defeated rats. Also, social defeat stress increased c-Fos expression in CeA and BNST of mice (Numa et al., 2019). In addition, lesions in CeA were able to alter behavioral and autonomic responses in stress condition (Roozendaal et al., 1991). Davis et al. (2010) suggested that the CeA is activated and can rapidly elicit stress-related responses immediately after a stressful event is detected. However, the BNST is activates in situations of chronic stress, fear, and anxiety.

Moreover, MS induced higher c-fos levels in BNST, BLA, CeA, and other limbic regions (Horii-Hayashi et al., 2012; Aguggia et al., 2013; Nishi et al., 2013). Interestingly, Jahng et al. (2010) showed that rats exposed to early-life MS presented a blunted accumbal Fos immunoreactivity response to restraint stress relative to controls. However, it remains unclear whether alcohol-induced neuronal activity in extended amygdala regions may differ between subjects that are exposed to stress in early-life and those that are not exposed, in a sex-dependent manner.

Taken together, these data show a complex relation between environmental experiences (including a history of early life stress), and biological factors, such as sex, on vulnerability to AUD. Although evidence points to the influence of MS stress on AUD development, the neurobiological mechanisms underlying this interaction remain unclear. Thus, we aimed to investigate the long-term effects of prolonged MS stress on alcohol-related behaviors in male and female adult mice. In addition, we evaluated the role of extended amygdala sub-nuclei in this phenomenon *via* the analysis of neuronal activity in those areas, in the different experimental groups. We hypothesized that MS would increase mice ethanol consumption and Fos-immunoreactive neurons (Fos-IR) in extended amygdala in a sex-dependent manner.

## Materials and Methods

### Ethics

The experimental protocols comply with the ethics principles of Brazilian legislation, which approves the use of animals for research (Conselho Nacional de Controle de Experimentação Animal – CONCEA) and were approved by the local ethics committee of Universidade Federal de São Paulo (Comissão de Ética no Uso de Animais – CEUA/UNIFESP). Approval protocol number 5360240918.

### Animals

Eight breeding pairs of C57BL/6J (20–30g, 10–12weeks) mice were obtained from Centro de Desenvolvimento de Modelos Experimentais para Biologia e Medicina – CEDEME (Universidade Federal de São Paulo/UNIFESP, São Paulo, SP, Brazil). The breeding pairs were housed in polycarbonate cages (29cm×18cm×13cm) and maintained under a reverse 12-h light cycle, as described below, in a temperature (23±2°C) and humidity-controlled environment. Animals had access to food and water *ad libitum* on a stainless-steel wire lid.

### Drug

Ninety Six percent ethanol (Synth, Diadema, Brazil) was diluted in filtered water at 20% (w/v); Quinine (Sigma-Aldrich, St. Louis, MO, United States) was dissolved in a 20% ethanol solution in increasing concentrations (0.005, 0.01, 0.025, and 0.05g/L).

### Maternal Separation Stress

Female mice were checked twice daily for newborn litters. The day of birth was considered PND 0. A total of 11 litters were used in this experiment: four litters for the control group (*n*=14; female=7, male=7), and seven for the MS group (*n*=14; female=7, male=7). Due to some litter problems, we needed to use more litters for MS group than control group. However, we understand the importance of equal distribution of litters between groups as described for Festing (2006) study. During the MS procedure, pups were removed from their nest for 3h/day, from PND 1 to PND 14. Litters were allocated to separate plastic containers lined with clean shavings. The containers were placed on a thermal blanket set at nest temperature (32–34°C), to prevent additional stress related to hypothermia. After 3h, the litters were returned to their parents in the nests. Pups from the control group were only handled during cage cleaning, which occurred twice a week. On PND 21, litters were weaned, and female and male littermates were housed separately in polycarbonate cages (up to four animals per cage). On PND 45, they were assigned to ethanol self-administration protocol.

### Ethanol Self-Administration Protocol

This protocol was based on the study of Blegen et al. (2018) and consists of two parts: (1) involuntary consumption and (2) operant ethanol self-administration. For this, female and male mice, whether submitted to MS stress or not, were allocated to a reversed light cycle room (lights on from 6:00pm to 6:00am), 7days before drinking experiments began.

#### Involuntary Consumption

On PND 45, in order to adapt to ethanol taste and assess its hedonic value, MS and control mice were placed in individual home cages, and water bottles were replaced with 50ml-plastic bottles containing 20% ethanol (w/v). 4h later, ethanol bottles were changed again for water bottles, and the animals were returned to their home cages. This procedure was repeated 5 consecutive days per week, over 3weeks. Ethanol bottles were fitted with stainless-steel sipper tubes with two ball-valve nipples to minimize spillage. Individual ethanol intake was determined by daily weighing of bottles, before and after the 4h of ethanol exposure. The consumption was corrected using a bottle that was identically handled and placed in an unoccupied cage for the same period of time. During this procedure, mice had free access to food. Ethanol solution was prepared by diluting 96% ethanol with filtered water to 20% (w/v).

#### Operant Ethanol Self-Administration

##### Apparatus

Operant chambers (170mm×150mm×200mm; Master-One, Ribeirão Preto, SP, Brazil) was equipped with a cup for fluid delivery in the center of the right wall, 45mm above the floor, connected to a syringe pump (Razel, Stamford®, CT, EUA). On both the left and right side of the chambers, nose poke sensors were mounted 45mm above the floor to detect nose entries. The active nose poke sensor allowed the animal to receive 0.1ml of 20% ethanol (w/v) reinforcement. This sensor activation is associated with discriminative cues, such as sound (900Hz; 20dB), and a red stimulus light (7.5W) above the nose poke, lasting 3s. After the reinforcement delivery, a 10-s “time out” period started. The number of pokes in the inactive nose poke sensor was recorded, but did not produce any responses. All sessions began with the white house light (7.5W) turned on. The devices in the operant chambers, as well as the pump, were controlled by a PC interface and AVS-PC Software. Ethanol intake was determined by subtracting the remaining solution in the cup at the end of the session from the volume dispensed into the cup.

##### Ethanol Self-Administration Training

To acquire ethanol self-administration in operant chambers, and also to assess ethanol hedonic value, mice were trained to self-administer 20% ethanol (w/v) in the operant chambers after 3weeks of involuntary consumption. All sessions lasted 2h/day, for 5 consecutive days per week (Monday to Friday). Initially, each nose poke response was reinforced with 0.1ml of ethanol solution (fixed ratio schedule of reinforcement: FR1). The following week, the requirement was increased to FR3 (three nose pokes were necessary to deliver a reinforcement with 0.1ml of ethanol solution) and in the last week it was increased to FR5 (five nose pokes were necessary to deliver a reinforcement with 0.1ml of ethanol solution).

##### Progressive-Ratio Schedule and Breaking Point

To assess the effort and the motivation to obtain reinforcement, MS and control female and male mice were challenged to self-administer a 20% ethanol solution on a progressive-ratio (PR) schedule, in which the number of nose-pokes required to earn each reinforcement was increased by three (i.e., 0, 3, 6, 9…). The last completed ratio, the number of active nose-pokes, and the total ethanol intake that resulted in the last reinforcement delivery was considered the breaking point. PR sessions were alternated with maintenance sessions, in which animals self-administered the ethanol solution on a FR5 schedule. In total, there were three PR sessions, alternated with two FR5 schedule sessions, over 1week. All sessions lasted 2h.

##### Binge Protocol

In this phase, MS and control female and male mice had access to 20% ethanol solution for 4h, on a FR5 reinforcement schedule.

##### Quinine Adulteration Tests

To assess the motivation and persistence of responding, in spite of quinine adulteration (aversive bitter taste), the 20% ethanol solution was adulterated with increasing concentrations of the bitter-tasting quinine (0.005, 0.01, 0.025, and 0.05g/L). Self-administration sessions lasted 2h/day on an FR5 reinforcement schedule. Each concentration was tested on a different day.

Twenty-four hours after the last quinine adulteration session, mice were submitted to a last operant-self administration session with 20% ethanol in a FR5 schedule. This session lasted 90min, and mice were immediately euthanized by perfusion for Immunohistochemistry procedure, as described below.

### Immunohistochemistry

The immunohistochemistry protocol was based in previous studies from our group (Cruz et al., 2014; Palombo et al., 2020; Felipe et al., 2021). About 90min after the beginning of the last operant self-administration session, all mice were anesthetized with Isoflurane (1/1ml – Isoforine, Cristália, Itapira, SP, Brazil) and perfused with 20ml of phosphate-buffered saline (PBS) followed by 60ml of 4% paraformaldehyde. Brains were post-fixed in 4% paraformaldehyde for 90min and transferred to 30% sucrose in PBS at 4°C for 3days. Brains were frozen in powdered dry ice and kept at −80°C until sectioning. Coronal sections of NAcc, BNST, and AMY were cut at 30μm using a cryostat (Leica CM1860 UV Cryostat; Leica Microsystems Inc., IL, United States). The sections were washed three times in phosphate buffer (PB) 0.1M, blocked with 3% normal goat serum (NGS) in PB 0.1M with 0.25% of Triton X-100, and incubated for 18h at 4°C with anti-Fos antibody (1:1,000, Cell Signaling, Danvers, MA, United States), diluted in blocking solution. Afterward, sections were washed in PB 0.1M, and incubated for 2h with biotinylated anti-rabbit secondary antibody (1:600, Vector Laboratories, Burlingame, CA, United States), in PB 0.1M with 0.25% of Triton X-100. After washing in PB 0.1M, sections were washed in PB 0.1M, and incubated for 90min in the avidin-biotin-peroxidase complex (ABC Elite kit, PK-610, Vector Laboratories) in PB 0.1M containing Triton X-100. Finally, sections were washed in PB 0.1M and incubated with 3,3'-diaminobenzidine for approximately 5min and washed five times. Then, they were transferred to PB 0.1M and mounted onto chrome alum-gelatin-coated slides. Once dry, they were dehydrated with a graded series of alcohol (Distilled water; alcohol 30, 60, 90, 95, and 100%) and Xilol before coverslipping with Entellan (Merck, Darmstadt, Germany). Images from Fos immunoreactivity in NAcc, BNST, and AMY were captured by a camera attached to a microscope (Zeiss Axio Imager D2®). Coordinates for Nacc: 1.18mm anterior to bregma, BNST: 0.14mm anterior to bregma, and AMY: 1.82mm posterior to bregma (Paxinos and Franklin, 2001). The images were analyzed by a blinded procedure.

### Statistical Analysis

Involuntary consumption, acquisition of self-administration and quinine adulteration test data were analyzed by Linear Mixed Model (LMM; within-subjects factor: time; between-subjects factor 1: sex; between-subjects factor 2: stress; random factor: subjects), followed by Bonferroni *post hoc* test for multiple comparison, where appropriate. The within-subjects factor “time” represents distinct meanings, depending on the protocol phase. For the involuntary consumption phase, “time” represents the 3 consecutive weeks in which the animals were submitted to the same procedure (i.e., time *per se*). For the self-administration phase, “time” represents the 3 consecutive weeks, in which the animals were submitted to distinct fixed ratios schedules (FR1, FR3, and FR5, respectively; i.e., time means the fixed ratios). For the quinine adulteration phase, “time” represents the increase in quinine concentration over the 4days of the protocol (i.e., time means quinine concentration). In addition, the choice of repeated covariance structure for the LMM was based on Akaike’s Information Criterion (AIC). We selected the most adequate structure (the one presenting the lowest AIC) after testing for the following covariance matrix: Diagonal, First-Order Autoregressive, Compound Symmetry, Scale Identity, or Unstructured.

The Progressive-Ratio schedule and Binge protocol were analyzed by Generalized Linear Model (GLM; between-subjects factor 1: sex; between-subjects factor 2: stress), followed by Bonferroni *post hoc* test for multiple comparison, where appropriate. The choice of data distribution and link function for the GLM was based on AIC. We selected the most adequate model (the one presenting the lowest AIC) after testing for the following distributions: Normal, Gamma, Poisson, or Negative Binomial. Subjects were considered a random factor as animals were not submitted to all the protocol phases equally. GLM was performed to analyze immunohistochemistry data. A significance level of 0.05 was set for all analyses.

### Experimental Design

**Figure 1:**
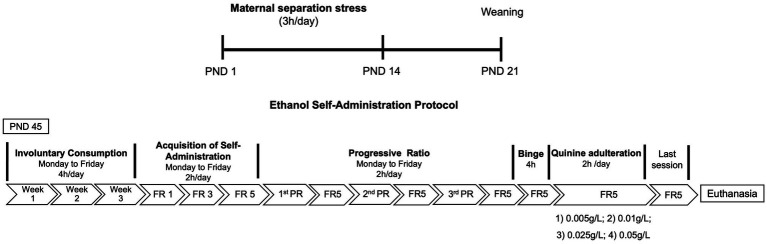
Experimental design. Pups (*n*=14; female=7, male=7) were separated from their dams 3h/day from postnatal day (PND) 1 to 14. Control mice *n*=14; female=7, male=7 were left undisturbed. On PND 45, mice were submitted to 4h of involuntary consumption during 3weeks. Afterward, mice underwent ethanol self-administration training in operant chambers under fixed ratio (FR) schedule. Sessions lasted 2h, 5days per week. Then, subjects were tested under 2h sessions of a progressive-ratio (PR) schedule of reinforcement (the last ratio achieved was considered the breaking point), and tested in a 4h session of binge drinking. After 24h, mice were submitted to a last operant session and were euthanized.

Female and male mice were subjected to MS stress, while mice from the control group were maintained with their dams in their home cages until PND 21, when the litters were weaned. On PND 45, ethanol self-administration experiments began. At the end, mice were perfused and their brains were processed to Immunohistochemistry protocol (Figure 1).

## Results

### Involuntary Consumption

**Figure 2:**
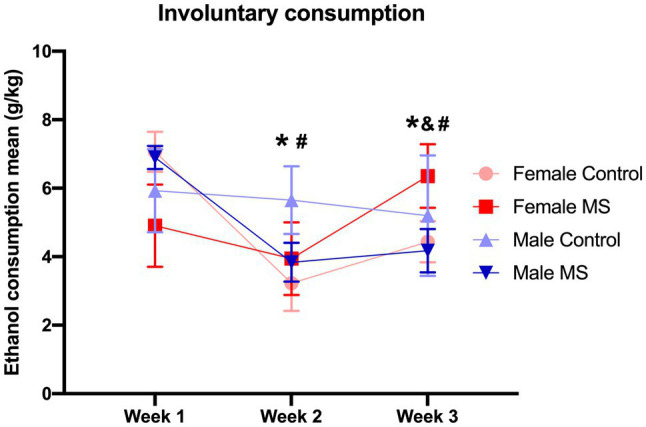
Involuntary consumption. Means of 20% ethanol consumption of female and male mice submitted to maternal separation (MS) and control group, during weeks 1, 2, and 3. *N*=28 (Control: *n*=14; female=7, male=7, MS: *n*=14; female=7, male=7). Linear mixed model (LMM) analysis, data: mean±SEM. ^*^p<0.05 female control group in weeks 2 and 3 compared to week 1; ^&^*p*<0.05 female MS group in week 3 compared to week 2; ^#^*p*<0.05 male MS group in weeks 2 and 3 compared to week 1.

Involuntary consumption (Figure 2, *n*=28) was recorded 5days per week, for 3weeks. Data were grouped weekly and are expressed as the mean ethanol consumption (g/kg of animal body weight). LMM analysis revealed an effect of time [*F*_(2,26.947)_=17.381, *p*<0.0001] and a triple interaction effect: sex*stress*time [*F*_(2,26.947)_=10.135, *p*<0.001]. In this sense, the female control group (*n*=7) consumed more ethanol in the first week of the protocol than in the following weeks, indicating that they reduced consumption over time. In the female MS group (*n*=7), the ethanol intake increased in the third week in comparison to the first and second weeks of the protocol, indicating that they increased consumption over time.

As with female control mice, the male MS group (*n*=7) decreased their consumption over time. In male control mice (*n*=7), ethanol intake remained the same during the 3weeks of protocol. LMM did not reveal any other main effect (sex or stress) or interaction effect (sex*stress, stress*time, or sex*time).

### Acquisition of Ethanol Self-Administration

**Figure 3:**
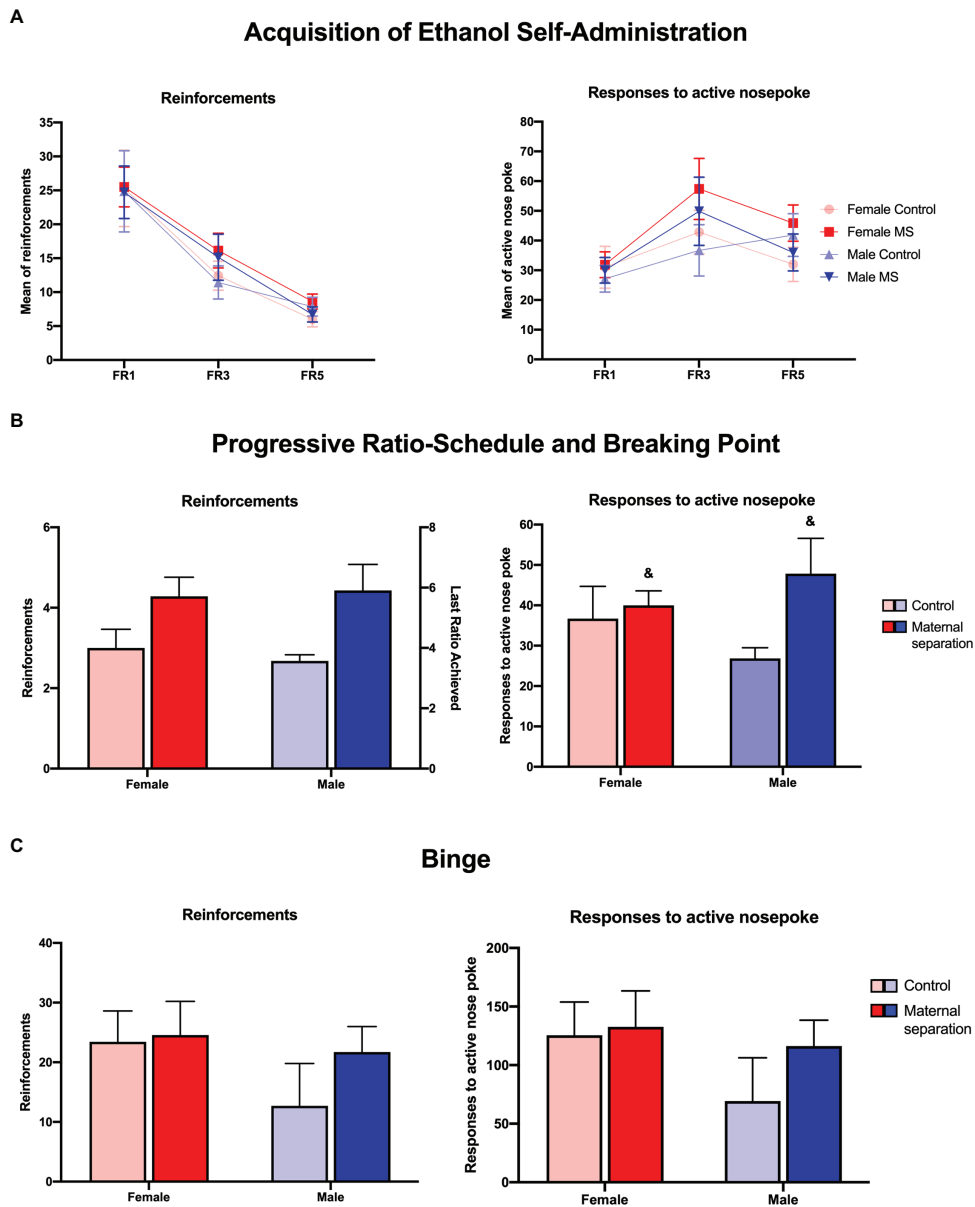
Operant self-administration protocol. **(A)** Means of reinforcements and responses to active nose poke of female and male mice submitted to MS and control group during FR1, FR3, and FR5 schedules of acquisition self-administration phase. **(B)** Means of reinforcements and highest completed ratio, mean of responses to active nose poke of female and male mice submitted to MS and control group during PR schedule. &MS stress>control, considering stress factor. **(C)** Means of reinforcements and mean of responses to active nose poke, of female and male mice submitted to MS and control group in Binge protocol. *N*=28 (Control: *n*=14; female=7, male=7, MS: *n*=14; female=7, male=7). LMM and generalized linear model (GLM) analysis, data: mean±SEM.

In the acquisition protocol for ethanol self-administration (Figure 3A), LMM analysis revealed an effect of fixed ratio for number of reinforcements [*F*_(2,35.154)_=45.106, *p*<0.0001], and number of responses to active nose pokes [*F*_(2,26.947)_=17.381, *p*<0.0001]. The number of reinforcements was higher in the FR1 week and decreased over the weeks (FR1>FR3>FR5), regardless of sex and neonatal handling. The opposite profile was observed for active nose poke, where the number of responses increased over time (FR1<FR3<FR5), regardless of sex and neonatal handling. No other effects were observed for all the variables analyzed. A total of 28 animals were used in this protocol phase (Control: *n*=14; female=7, male=7 and MS *n*=14; female=7, male=7).

### Progressive-Ratio Schedule and Breaking Point

In the Progressive-Ratio protocol (Figure 3B), GLM analysis did not reveal any difference for the number of reinforcements. Regarding active nose poke, GLM analysis indicated an effect of stress [W*χ*^2^(1)=4.307, *p*=0.038]. In this sense, mice submitted to MS stress (*n*=14; female=7, male=7) presented more responses to active nose pokes than mice in the control group (*n*=14; female=7, male=7), regardless of sex. GLM did not reveal a main effect of sex, nor a sex*stress interaction effect.

### Binge Protocol

In binge protocol for ethanol self-administration, GLM analysis showed no effect of sex, stress, or sex*time interaction for either of the ethanol self-administration variables analyzed (Figure 3C). A total of 28 animals were used in this protocol phase (Control: *n*=14; female=7, male=7 and MS *n*=14; female=7, male=7).

### Quinine Adulteration Test

**Figure 4:**
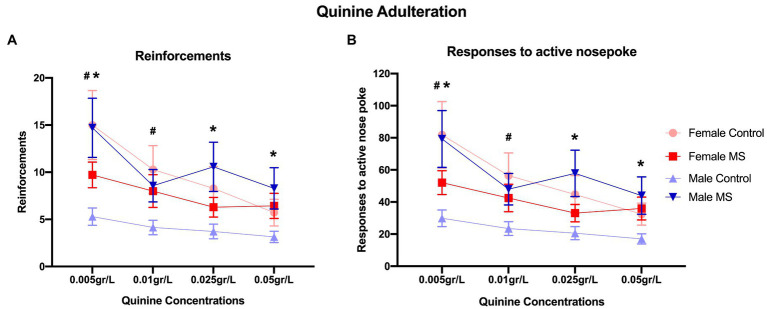
Quinine adulteration protocol. Means of reinforcements **(A)** and mean of responses to active nose poke **(B)** of female and male mice submitted to MS and control group. *N*=28 (Control: *n*=14; female=7, male=7, MS: *n*=14; female=7, male=7). LMM analysis, data: mean±SEM. ^*^p<0.05 male MS group compared to male control group; ^#^*p*<0.05 female control group reinforcements and responses were higher in concentrations 0.005 and 0.01g/L compared to concentration 0.05g/L.

In the quinine adulteration protocol for ethanol self-administration (Figure 4), LMM analysis revealed an effect of quinine concentration for the number of reinforcements [*F*_(3,33.573)_=12.425, *p*<0.0001] and responses to active nose pokes [*F*_(3, 34.976)_=11.048, *p*<0.0001]. For both variables (number of reinforcements and active nose pokes) the lowest quinine concentration (0.005g/L) presented the highest number of responses, while the other concentrations (0.01, 0.025, and 0.05g/L) presented a similar number of responses.

Linear mixed model analysis also revealed a sex*stress interaction effect for the number of reinforcements [*F*_(1,25.339)_=7.416, *p*=0.012] and active nose pokes [*F*_(1, 24.304)_=7.425, *p*=0.012]. For both variables (number of reinforcements and active nose pokes) control (*n*=7) and MS (*n*=7) female groups showed similar behavior, while male control animals (*n*=7) presented a lower number of responses and reinforcement when compared to the MS group (*n*=7). Moreover, male control animals also presented a lower number of responses and reinforcement than female control, while no differences were found in responses to quinine taste between female and male offspring submitted to MS stress maternal separation.

In addition, a triple interaction effect (sex*stress*quinine concentration) was observed for both the number of reinforcements [*F*_(3,33.573)_=3.326, *p*=0.031] and active nose pokes [*F*_(3, 34.976)_=3.199, *p*=0.035]. The number of reinforcements decreased over time for female control animals (0.005 and 0.01g/L>0.05g/L; also 0.005g/L>0.025g/L) and for male MS animals (0.005g/L>0.01 and 0.05g/L). No differences were observed for male control animals and for female MS groups. Moreover, the number of reinforcements in 0.005 and 0.01g/L was higher for the female control group than for the male control. Similarly, the number of reinforcements in 0.005, 0.025, and 0.05g/L was higher for male MS offspring than for male control. Regarding the number of active nose pokes, similar results were found, except for the male MS group (0.005g/L>0.05g/L but 0.005g/L=0.01g/L).

### Immunohistochemistry for Fos-Immunoreactive Neurons

**Figure 5:**
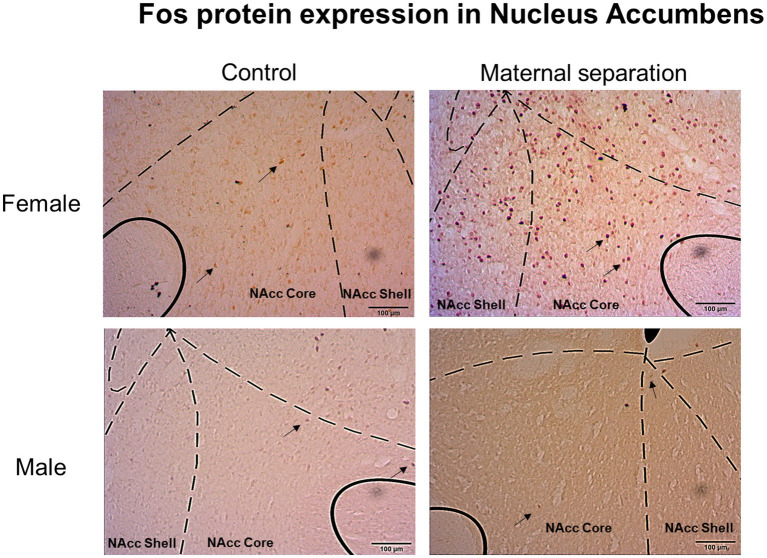
Fos protein expression in nucleus accumbens (Nacc) Core and Shell. Images represent Fos positive cells in Nacc Core and Shell of female and male mice submitted to MS and control group. Black arrows indicate Fos-immunoreactive neurons (Fos-IR) neurons. Coordinates for Nacc: 1.18mm anterior to bregma (Paxinos and Franklin, 2001).

**Figure 6:**
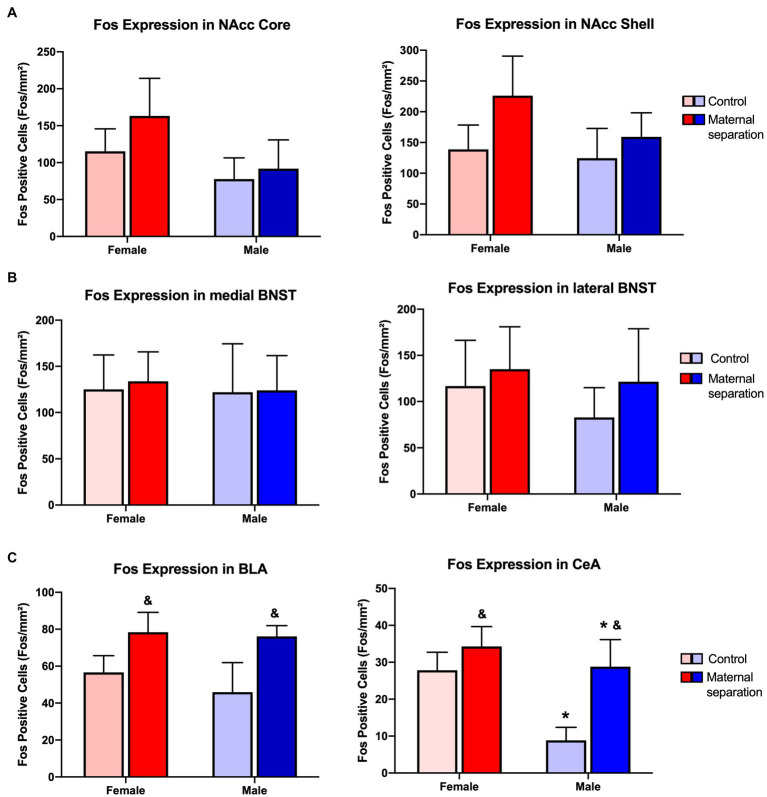
Immunohistochemistry assay for Fos protein. Number of Fos-IR neurons presented as Fos positive cells per mm^2^ in Nacc Core and Shell **(A)**, medial and lateral BNST **(B)**, and BLA e central amygdala (CeA; **C**). *N*=6–7/group. GLM analysis, data: mean±SEM. &: MS stress>control, considering stress factor. *: male<female, considering sex factor.

**Figure 7:**
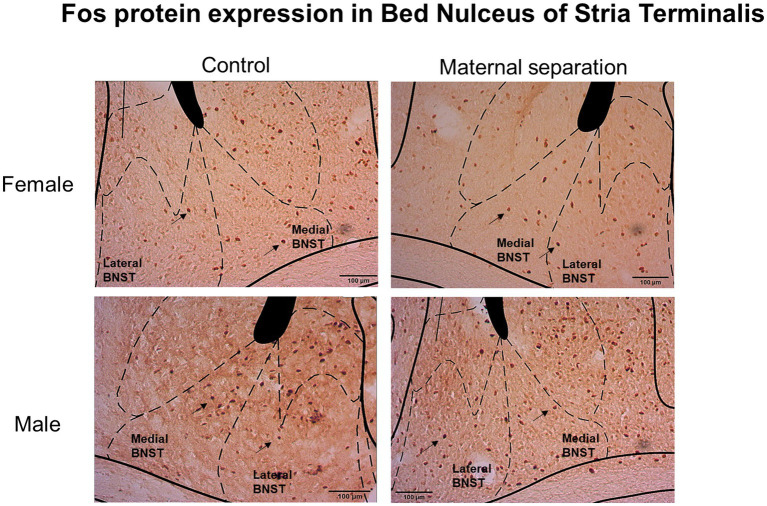
Fos protein expression in medial and lateral bed nucleus of stria terminalis (BNST). Images represent Fos positive cells in medial and lateral BNST of female and male mice submitted to MS and control group. Black arrows indicate Fos-IR neurons. Coordinates for BNST: 0.14mm anterior to bregma (Paxinos and Franklin, 2001).

Considering the number of Fos-IR neurons in Nucleus Accumbens Core and Shell regions (Figure 5), GLM analysis did not show any effect of sex, stress, or sex*stress interaction (Figure 6A). In addition, GLM did not reveal any effect of sex, stress, or sex*stress interaction for the number of Fos positive (Fos+) cells in both the lateral and medial BNST subdivisions (Figures 7 and 6B).

**Figure 8:**
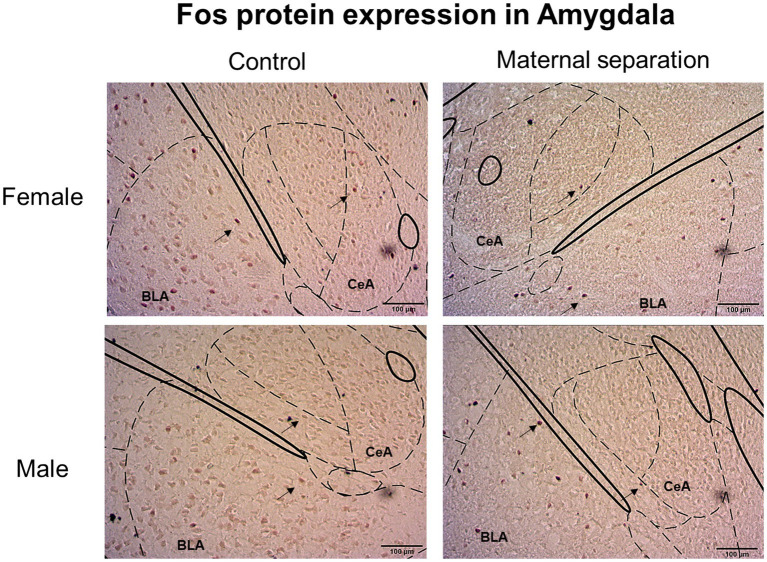
Fos protein expression in BLA and CeA. Images represent Fos positive cells in BLA and CeA of female and male mice submitted to MS and control group. Black arrows indicate Fos-IR neurons. Coordinates for amygdala (AMY): 1.82mm posterior to bregma (Paxinos and Franklin, 2001).

In Basolateral Amygdala (Figure 8), the effect of stress was observed [W*χ*^2^(1)=6.646; *p*=0.010]. In this regard, the number of Fos-IR neurons in the BLA of mice submitted to MS stress was higher than the control group, regardless of sex (Figure 6C).

For CeA (Figure 8), GLM showed a sex factor effect [W*χ*^2^(1)=5.982; *p*=0.014]; regardless of stress factor, the number of Fos-IR cells in this brain region of female mice was higher than the male groups. Also, stress factor was observed [W*χ*^2^(1)=6.998; *p*=0.008], and mice from MS groups presented more Fos+ neurons than the control groups (Figure 6C).

## Discussion

Given the importance of environmental experiences and biological factors as predictors of ethanol consumption, we investigated the influence of MS stress on ethanol consumption in female and male adult mice, and the participation of the extended amygdala in this interaction.

We showed that during involuntary consumption, MS increased ethanol intake in female mice, while it decreased consumption in males. In addition, regardless of sex, animals submitted to early life stress were more motivated to seek ethanol in a progressive-ratio schedule of operant self-administration procedure. Moreover, female controls, as well as male MS mice, were more resistant to decreasing their consumption relative to male control mice during the quinine adulteration protocol. For immunohistochemistry, we observed an increase of Fos-immunoreactive neurons in the BLA and CeA in MS mice, but not in NAcc, nor in BNST.

Interestingly, during the involuntary consumption paradigm, female controls and MS males presented a similar pattern of consumption, showing a decrease in consumption during the second and third weeks of the procedure. In contrast, male controls exhibited a linear consumption pattern over the weeks, while MS females increased their intake during the third week relative to the second week. Although no statistical differences were found between MS and control or male and female groups, we observed a possible effect of MS stress on ethanol consumption in a sex-dependent model, as the four experimental groups presented distinct ethanol intake patterns over the weeks.

Concerning human clinical studies, women and men differ significantly in the development of AUD (Haas and Peters, 2000). Although women initiate alcohol use later in life than men, they show progress faster from drug use to abuse, especially when using psychostimulants, such as cocaine (Haas and Peters, 2000; Castro-Zavala et al., 2020).

Studies suggest that females are more susceptible to stress-potentiated ethanol consumption, to alleviate the negative stress effects and reactivity (Armstrong et al., 2018; Peltier et al., 2019). In addition, Kawakami et al. (2007) have demonstrated that after chronic exposure to ethanol, female mice submitted to long periods of MS showed faster development of behavioral sensitization when compared to male mice. In addition, Lancaster (1998) reported that female rats submitted to short periods of MS, from PND 1 to 7, increased ethanol intake. These findings agree with our results showing that stressed female mice increased their consumption over time. On the other hand, Castro-Zavala et al. (2020) demonstrated that female rats submitted to early-life stress decreased self-administration of a psychostimulant drug in adulthood.

In contrast with many studies that reported increased ethanol intake of male rodents after exposure to different stressors (Nash and Maickel, 1985; Lynch et al., 1999; Wille-Bille et al., 2017; Newman et al., 2018), including MS stress in which male rats were submitted to 360min MS stress, from PND 1 to PND 21 (Roman et al., 2005), we observed a decrease in ethanol intake of male MS mice. This kind of stress may have induced an anhedonic effect in male mice, which could lead to reduced intake. Willner et al. (1992) suggested that chronic exposure to a variety of mild stressors may cause a decreased responsiveness to reward, when rats were exposed to a sequence of different and unpredictable stressors from 5 to 9weeks, reducing the consumption and preference for saccharin or sucrose solutions (Willner et al., 1987). In the same way, Huot et al. (2001) showed that maternally-separated rats exhibited anhedonia, as they avoided sucrose solution.

Regarding the self-administration protocol in fixed-ratio schedules, we did not observe a MS effect for male or female mice. To the best of our knowledge, there are few studies using the ethanol self-administration approach in mice. For instance, Cruz et al. (2008) demonstrated that maternally-separated male mice separated from their dams during 180min from PND 1 to PND 14, presented an increased consumption of 6 and 10% ethanol+saccharin solutions. Although these data differ from ours, it is important to highlight at least three major protocol differences between the Cruz study and ours: (i) unlike that study, our protocol included a 3-week-ethanol exposure prior to operant tasks. (ii) Cruz et al. (2008) added saccharin to ethanol solutions to augment response levels, which may have increased the hedonic value assigned to the substance, leading to a different outcome. (iii) In their study they used Swiss mice, while in ours we used C57BL/6J, which have shown a higher ethanol intake compared to other strains (Rhodes et al., 2007). Therefore, the control and MS mice could have reached the ceiling effect of consumption and this could have impaired the MS stress effects.

We did not detect a sex effect, when comparing male vs. female MS groups. In agreement with our findings, Logrip and Gainey (2020) showed that previous footshock stress did not alter the acquisition of ethanol self-administration behavior of male and female rats on FR1 and FR3 schedules. Conversely, female rats exposed to predator odor stress increased responses to ethanol self-administration in comparison to stressed male rats (Ornelas et al., 2021). Regarding undisturbed control groups, we also did not observe a sex effect. In contrast, in a FR4 schedule, male rats exhibited a higher ethanol self-administration in comparison to females (Lorrai et al., 2019). In addition, Randall et al. (2017) showed that male rats presented increased operant responses for ethanol+sucrose solution relative to female rats.

The progressive-ratio schedule has been widely used to evaluate motivation and goal-directed behavior toward rewards (Randall et al., 2017). Our data reveal that regardless of sex, MS increased the motivation to take ethanol reinforcement, considering the number of active nose pokes.

Few studies have evaluated early life stress effects on motivation for ethanol, especially in mice, and the data is contradictory in the literature. Fosnocht et al. (2019) demonstrated that prolonged social isolation during adolescence increased the motivation of male and female adult mice to seek cocaine, in a progressive-ratio schedule. However, Wistar rats submitted to early pup-litter-dam deprivation exhibited reduced motivation to obtain sucrose in the same paradigm (Rüedi-Bettschen et al., 2005). Based on our data, we suggest that MS altered the motivation of mice to seek ethanol, but not necessarily hedonic value of this substance under a self-administration paradigm, since both MS and control groups presented similar numbers of reinforcements and active nose poke responses during FR tests. Furthermore, progressive-ratio experiments assessing the influence of MS on motivation toward other drugs of abuse and natural reinforcers, such as sucrose, would contribute to a better understanding of this effect.

During the binge test, we did not observe sex differences in relation to the number of reinforcements or active responses. However, Finn et al. (2018) showed that intermittent predator odor stress exposure increased ethanol binge drinking in male mice, but did not alter drug consumption in females. On the other hand, Sneddon et al. (2018) demonstrated that female mice increased binge consumption, while males did not. It is important to highlight that in these studies, binge drinking was not evaluated through an operant behavior, but by measuring ethanol consumption in home cages. Also, mice were exposed to ethanol consumption for 7weeks in our study, to acquire the operant behavior while in both the Finn and Sneddon studies, the animals were submitted to ethanol intake for up to 3weeks.

In regard to stress effect, we also did not observe differences between MS and control groups. Nevertheless, Gondré-Lewis et al. (2016) showed that MS stress increased response to ethanol during a binge session of self-administration protocol in rats. However, there are some aspects of Gondré-Lewis protocol that are worth mentioning: the multiple consecutive binge sessions (with no previous abstinence) were constituted by two interspersed 45min-periods, and sucrose was added to the ethanol solution. In comparison, in the present study the single binge session lasted 4h and followed a 2-day abstinence period, which we hypothesized may have influenced the control groups intake.

In the quinine adulteration protocol, we observed that female control mice presented increased ethanol intake relative to male controls for 0.005 and 0.01g/L quinine concentrations. This result suggests that females are more resistant to extinguishing or decreasing ethanol-seeking behavior than male mice when presented with an aversive stimulus.

Consistent with our findings, Sneddon et al. (2020) demonstrated that in an operant self-administration paradigm, male mice reduced their response to adulterated ethanol solution in high quinine concentrations, but female mice did not exhibit changes in their response to adulterated ethanol at any concentration. Furthermore, Fulenwider et al. (2019) showed that a higher concentration of quinine was required to suppress the ethanol consumption of female mice compared to male mice. In addition, the Sneddon group (2018) showed that with the two-bottle choice of access in the “Drinking in the Dark” protocol, female mice responded more to quinine-adulterated ethanol solution than male mice, but high concentrations of quinine suppressed the consumption of both male and female mice.

In relation to MS stress, we observed that only male mice exhibited increased adulterated ethanol solution intake relative to the male control group. In this sense, male mice subjected to chronic predator-exposure stress consumed more quinine-adulterated ethanol solution than non-stressed animals, and they only exhibited a reduction in ethanol intake when quinine reached the highest concentration (Shaw et al., 2020). Nevertheless, female rats submitted to acute early life stress (footshock) tolerated higher quinine concentrations compared to their control group. Male control rats were more sensitive to quinine taste, and the consumption of the stressed male group decreased only with the highest quinine concentration (Radke et al., 2019).

Results from this trial may suggest that the male MS group are more resistant to bitter quinine taste than its control group. Also, the responses to the aversive taste of quinine seems to be related to a number of experimental variables, such as type and duration of stress, quinine concentration, and the species (rats vs. mice).

Apparently, this is the first study to evaluate extended amygdala neuronal activation of mice that have been chronically exposed to both MS and ethanol in a sex-dependent model.

As described in the immunohistochemistry procedure, male and female mice were submitted to a last operant self-administration session before brain collection. This session followed the quinine adulteration protocol, and animals were allowed to consume 20% ethanol after a period of an aversive stimulus. In this sense, this last operant session may have contributed to Fos protein expression in neurons of the NAcc core and shell of male and female mice from MS and control groups.

Our results did not show any differences in Fos-IR cells of NAcc core and shell subregions among MS and control mice groups (male or female). In the same sense, Saalfield and Spear (2019) observed that after administration of two different concentrations of ethanol, adult rats did not show any differences of Fos positive cells in NAcc core. However, Leriche et al. (2008) showed that ethanol intragastric administration increased the number of Fos-immunoreactive neurons in NAcc shell of rats. Ethanol intraperitonial injection also increased the number of Fos positive cells in this same brain region (Herring et al., 2006). Further, Ryabinin et al. (1999) observed an increased number of Fos-IR neurons in the NAcc Core, but not in the NAcc shell in mice submitted to restraint stress after ethanol consumption. It is important to highlight that in these studies, ethanol administration was not evaluated through operant behavior, such in the present study. Moreover, our study assessed Fos expression 24h after the quinine adulteration protocol, an aversive stimulus. This may also have contributed to difference in the results.

We did not observe any differences in Fos protein expression in neurons of medial- and lateral-BNST subdivisions in female and male mice of control or MS groups. However, Ryabinin et al. (1999) showed that restraint stress increased the number of Fos+ cells in the BNST of mice chronically exposed to ethanol. In fact, the stressful stimulus exposure occurred after ethanol consumption, and not during the early life of the mice.

Although, we did not find any differences in neurons expressing Fos protein between MS and control groups in the BNST, we observed increased neuronal activation in the Central and Basolateral Amygdala.

In the BLA, we observed the effect of stress, and the Fos protein expression was higher in MS groups when compared to control groups. In agreement with our results, it has been demonstrated that MS stress increases the number of Fos+ cells in the BLA of male mice (Horii-Hayashi et al., 2012; Nishi et al., 2013). However, this increased Fos expression occurred on PND 14, after the final separation, but not during adulthood.

In the Central Amygdala, we observed that Fos expression of female mice was higher than in the male group. This may be due to the increased sensitivity to ethanol inhibition that male mice CeA neurons show compared to female mice CeA neurons (Logrip et al., 2017).

We also found that the number of Fos-IR neurons in the CeA of MS groups was higher than in control groups. An important integrative role between stress- and addiction-related behaviors has been attributed to the CeA (Gilpin, 2012). In relation to this, Horii-Hayashi et al. (2012) demonstrated that MS stress exposure alters CeA activation in male mice and increases Fos expression in this brain region. On the other hand, this amygdalar nucleus is also involved in ethanol response behaviors. For instance, acute ethanol exposure can increase the number of Fos-immunoreactive neurons in the CeA (Leriche et al., 2008; Sharko et al., 2016). In addition, De Guglielmo et al. (2016) demonstrated that the inactivation of a CeA neuronal ensemble during abstinence decreased ethanol consumption of dependent and non-dependent rats.

Therefore, we hypothesize that MS stress could induce neuroadaptations in the CeA, which are related to increased neuronal activation and ethanol consumption. Nonetheless, future studies assessing the effects of activation or inhibition of CeA on ethanol consumption of animals exposed to early life stress, such as MS are needed to corroborate our hypothesis.

As future perspectives, studies aimed at investigating which CeA neuroadaptations are involved in both stress and ethanol consumption responses will help to understand this complex interaction better.

In conclusion, MS stress produces long-term alterations in the ethanol consumption of adult mice and BLA and CeA activation patterns, depending on reinforcement protocol and sex.

## Data Availability Statement

The raw data supporting the conclusions of this article will be made available by the authors, without undue reservation.

## Ethics Statement

The animal study was reviewed and approved by Comissão de Ética no Uso de Animais (CEUA/UNIFESP) n. 5360240918.

## Author Contributions

FC, TM, and NB contributed to the conception and design of the study. NB, CF, PP, TY, TR, and BT performed the experiments. NB and CL performed the statistical analysis. NB wrote the first draft of the manuscript. FC, TM, RL, CF, CL, and NB contributed to manuscript revision and read and approved the submitted version. All authors contributed to the article and approved the submitted version.

## Funding

The study was supported by Coordenação de Aperfeiçoamento de Pessoal de Nível Superior (CAPES) and Fundação de Amparo à Pesquisa do Estado de São Paulo (FAPESP). CL is recipient of Coordenação de Aperfeiçoamento de Pessoal de Nível Superior (CAPES) research fellowship through Instituto Nacional de Ciência e Tecnologia Translacional em Medicina (INCT-TM), Brazil. TY and TR are recipient of Coordenação de Aperfeiçoamento de Pessoal de Nível Superior (CAPES) research fellowship. NB is recipient of Fundação de Amparo à Pesquisa do Estado de São Paulo (FAPESP) fellowship (process number 2020/04389-3). FC is recipient of a FAPESP grant number 2018/15505-5.

## Conflict of Interest

The authors declare that the research was conducted in the absence of any commercial or financial relationships that could be construed as a potential conflict of interest.

## Publisher’s Note

All claims expressed in this article are solely those of the authors and do not necessarily represent those of their affiliated organizations, or those of the publisher, the editors and the reviewers. Any product that may be evaluated in this article, or claim that may be made by its manufacturer, is not guaranteed or endorsed by the publisher.
